# Clinical characteristics, molecular mechanisms, and exploration of association with gastrointestinal symptoms in *CHAMP1* gene variation-related neurodevelopmental disorders

**DOI:** 10.3389/fneur.2025.1664776

**Published:** 2025-10-02

**Authors:** Ziming Xu, Yan Xu, Xiaoyou Tao, Chen Chen, Guojuan Dong

**Affiliations:** ^1^Henan University of Chinese Medicine, Zhengzhou, Henan, China; ^2^Changchun University of Chinese Medicine, Changchun, Jilin, China

**Keywords:** *CHAMP1* gene, CHAND, truncating mutation, developmental delay, gastrointestinal symptoms, haploinsufficiency, dominant-negative effect, kinetochore-microtubule attachment

## Abstract

The *CHAMP1* (Chromosome Alignment-Maintaining Phosphoprotein 1) gene encodes a nuclear protein crucial for maintaining proper chromosome alignment and genomic stability during cell mitosis. Heterozygous variants of this gene, particularly *de novo* truncating mutations, are the primary cause of a rare neurodevelopmental disorder: autosomal dominant intellectual disability Autosomal Dominant Mental Retardation 40 (MRD40) or *CHAMP1*-related Neurodevelopmental Disorder (CHAND). The core clinical features of this disorder include moderate to severe global developmental delay, intellectual disability, significant language impairment, and distinctive facial features. Additionally, patients may exhibit abnormal muscle tone, behavioral issues (such as autism spectrum disorder traits and attention deficit hyperactivity disorder), epilepsy, microcephaly, and involvement of other multi-systemic complications, including gastrointestinal dysfunction. The pathogenic mechanisms of *CHAMP1* truncating mutations remain debated, with main hypotheses including haploinsufficiency and dominant-negative effect or gain-of-function, where the latter better explains the more severe clinical phenotypes observed in some patients. Although neurological manifestations are the research focus of *CHAMP1*-related disorders, the involvement of other systems such as the digestive system—particularly symptoms like repeated vomiting—has been underreported and lacks systematic research within this disease spectrum. This review aims to integrate the latest research progress on the molecular functions of the *CHAMP1* gene, the pathogenic mechanisms of its variants, and the clinical phenotype spectrum of related neurodevelopmental disorders. Based on clinical observations, we also preliminarily explored the potential association between *CHAMP1* gene variation and gastrointestinal symptoms (especially recurrent vomiting), with the goal of providing valuable references for clinical diagnosis, management, and future research directions for this rare disease.

## 1 Introduction

### 1.1 Molecular biology and cellular functions of the *CHAMP1* gene

*CHAMP1* (Chromosome Alignment-Maintaining Phosphoprotein 1), also referred to as *CAMP* in early literature (gene aliases: *C13orf8* or *ZNF828*), encodes a highly conserved nuclear phosphoprotein containing multiple zinc finger domains (1). This protein is dynamically expressed during the cell cycle and highly phosphorylated during mitosis, with its normal function being essential for cell proliferation and differentiation. The *CHAMP1* protein contains characteristic domains, such as the N-terminal WK motif, SPE motif, and FPE motif, as well as multiple C2H2-type zinc finger domains at the C-terminus. These domains endow *CHAMP1* with specific cellular localization and functions. Studies show that the *CHAMP1* protein primarily localizes to the nucleus and specifically accumulates at chromosomes, kinetochores, and spindles during mitosis (1).

The core cellular function of *CHAMP1* lies in precisely regulating the correct attachment of kinetochores to microtubules during mitosis, thereby ensuring faithful chromosome segregation into daughter cells (1). Loss of *CHAMP1* function leads to severe chromosome misalignment and mitotic arrest. Recent studies have further revealed broader roles of *CHAMP1* in cellular processes. *CHAMP1* and its protein complexes (e.g., the *CHAMP* complex containing *POGZ* and *HP1α*) are involved in heterochromatin assembly and maintenance (2). Additionally, *CHAMP1* plays a critical role in the homologous recombination repair (Homology-Directed Repair, HDR) pathway of DNA double-strand breaks (Double-Strand Breaks, DSBs), promoting DNA damage repair through interactions with proteins like *REV7*, thus maintaining genomic integrity and stability (2–4). *CHAMP1* also recruits the methyltransferase *SETDB1* to heterochromatic regions to regulate gene expression at specific loci (2). Through complex interaction networks with *POGZ*, *HP1α*, *REV7*, *CDC16*, *UPF3*, and other potential proteins, *CHAMP1* executes pleiotropic functions in cell division, genome maintenance, and gene expression regulation (1, 2, 5, 6).

### 1.2 *CHAMP1* gene mutations: from rare diseases to core genes of neurodevelopmental disorders

Around 2015, large-scale parallel sequencing technologies—particularly whole-exome sequencing of sporadic intellectual disability/developmental delay cohorts—first revealed that *de novo* heterozygous mutations in *CHAMP1* are associated with severe neurodevelopmental disorders (5, 7, 8). Early studies found that most pathogenic variants in *CHAMP1* are protein-truncating variants (e.g., nonsense mutations or frameshift mutations), which are extremely rare or absent in healthy population databases.

Subsequent studies rapidly accumulated more cases, establishing *CHAMP1* gene variants as the molecular etiology of an independent clinical syndrome: autosomal dominant intellectual disability type 40 (MRD40), broadly termed CHAND (9, 10). The reported *CHAMP1* gene c.1908C > G (p. Y636*) variant belongs to this common type of pathogenic truncation mutation that causes protein premature termination, and this particular variant has been shown to cause MRD40 (9). This discovery transformed *CHAMP1* from a gene primarily studied in cell biology to a core pathogenic gene of clinical diagnostic significance in neurogenetics and pediatrics.

### 1.3 Research status and focus of this review

Since the pathogenicity of *CHAMP1* was identified, academia has continuously explored the clinical phenotype spectrum and molecular mechanisms of related neurodevelopmental disorders. Current understanding of CHAND/MRD40 primarily focuses on core neurological manifestations, including intellectual disability of varying severity, global developmental delay (particularly significant delays in language and motor development), distinctive facial features, and neuropsychiatric behaviors (e.g., autism spectrum disorder [ASD] traits, attention deficit hyperactivity disorder [ADHD], stereotypic behaviors) (10, 11). Brain imaging may show nonspecific structural abnormalities.

However, with increasing case reports, the phenotypic spectrum of CHAND has expanded. In addition to core neurological impairments, involvement of other systems—including growth retardation, skeletal abnormalities, ophthalmological issues, and gastrointestinal dysfunction—has gained attention (10, 11). However, in particular, the prevalence of digestive symptoms (repeated vomiting) in this spectrum of diseases, clinical characteristics, and relationship with *CHAMP1* variants remains limited. Moreover, academic debates persist regarding the specific molecular mechanisms of different *CHAMP1* variant types (e.g., truncating mutations, missense mutations, gene deletions) and their impacts on clinical phenotype severity and specificity.

This review systematically summarizes the latest progress on *CHAMP1*’s molecular functions, variant pathogenic mechanisms, and clinical phenotype spectra of CHANDs. It specifically focuses on and preliminarily explores the potential association between *CHAMP1* variants and gastrointestinal symptoms (especially repeated vomiting), combine the existing literature evidence for analysis. Through in-depth discussions, we aim to provide insights for clinical diagnosis, patient management, and future research on *CHAMP1*-related rare diseases.

## 2 Molecular pathogenic mechanisms of *CHAMP1* gene mutations

### 2.1 Variant spectrum of the *CHAMP1* gene

Pathogenic variants in *CHAMP1* predominantly occur as heterozygous *de novo* events, following an autosomal dominant inheritance pattern where a single allele variant suffices to induce disease phenotypes of CHAND/MRD40 (5, 7). Most reported pathogenic variants are truncating variants (nonsense mutations or frameshift mutations) that introduce premature termination codons in the protein-coding sequence (6, 11). These truncating variants are widely distributed across the *CHAMP1* coding region but often occur before the C-terminal zinc finger domains. For example, the p. Y636* variant in our focus case is a typical example, confirmed in literature to be associated with MRD40 (9).

Besides truncating variants, missense variants and chromosomal microdeletions/duplications involving the *CHAMP1* locus have been reported in a minority of patients. The specific pathogenic roles and clinical significance of these non-truncating variants are still under investigation, with some missense variants potentially linked to atypical or more severe phenotypes (e.g., early-onset epileptic encephalopathy) (6).

### 2.2 Pathogenic mechanisms of truncating mutations: haploinsufficiency or dominant-negative effect?

The exact molecular mechanisms of *CHAMP1* truncating mutations remain debated, with two main hypotheses: haploinsufficiency and dominant-negative effect or gain-of-function.

Early theories proposed that truncating mutations induce haploinsufficiency via nonsense-mediated mRNA decay (NMD), reducing mutant allele mRNA levels or producing unstable/truncated proteins lacking critical domains, thereby halving functional *CHAMP1* protein levels (3). *CHAMP1* dosage is critical for cellular functions (e.g., DNA repair, chromosome segregation), and insufficient dosage triggers pathophysiological changes leading to neurodevelopmental disorders. A study analyzing lymphoblasts and fibroblasts from patients with premature termination codon (PTC) mutations detected truncated *CHAMP1* proteins and observed HDR pathway defects after induced DSBs, supporting loss-of-function via haploinsufficiency (3).

However, recent studies challenge the sole haploinsufficiency model. Clinical phenotype comparisons between patients with large *CHAMP1* deletions (true haploinsufficiency) and those with intragenic truncating mutations (e.g., PTC) showed that deletion carriers had milder phenotypes (e.g., borderline intelligence or mild developmental delay) with less impact on quality of life, whereas truncating mutation carriers exhibited typical moderate-to-severe intellectual disability and other neurodevelopmental issues (6, 11). This suggests that truncated proteins from PTC mutations may interfere with wild-type *CHAMP1* function via dominant-negative effects or acquire toxic gain-of-function properties. For example, truncated proteins may retain partial binding domains, causing erroneous protein interactions or mislocalization and disrupting cellular signaling pathways. Understanding the molecular consequences of truncating mutations (e.g., p. Y636*)—whether they primarily reduce protein levels or produce interfering truncated proteins—is crucial for prognosis and therapy development.

Cytological studies have shown that *CHAMP1*-deficient cells exhibit reduced DNA repair capacity (particularly impaired HDR), abnormal chromosome segregation, and multipolar spindle formation, which are pathological bases for neuronal developmental and functional abnormalities (2).

### 2.3 Mechanisms of other variant types

Missense variants in *CHAMP1* may have more complex pathogenic mechanisms. Some may disrupt specific functional domains (e.g., zinc finger domains or protein interaction interfaces), affecting molecular binding or enzymatic activity (if applicable). Reports link certain missense variants to severe epileptic encephalopathies with early-onset refractory epilepsy, suggesting gain-of-function mechanisms where mutant proteins acquire abnormal or hyperactive functions disrupting neural development (6).

**Figure 1:**
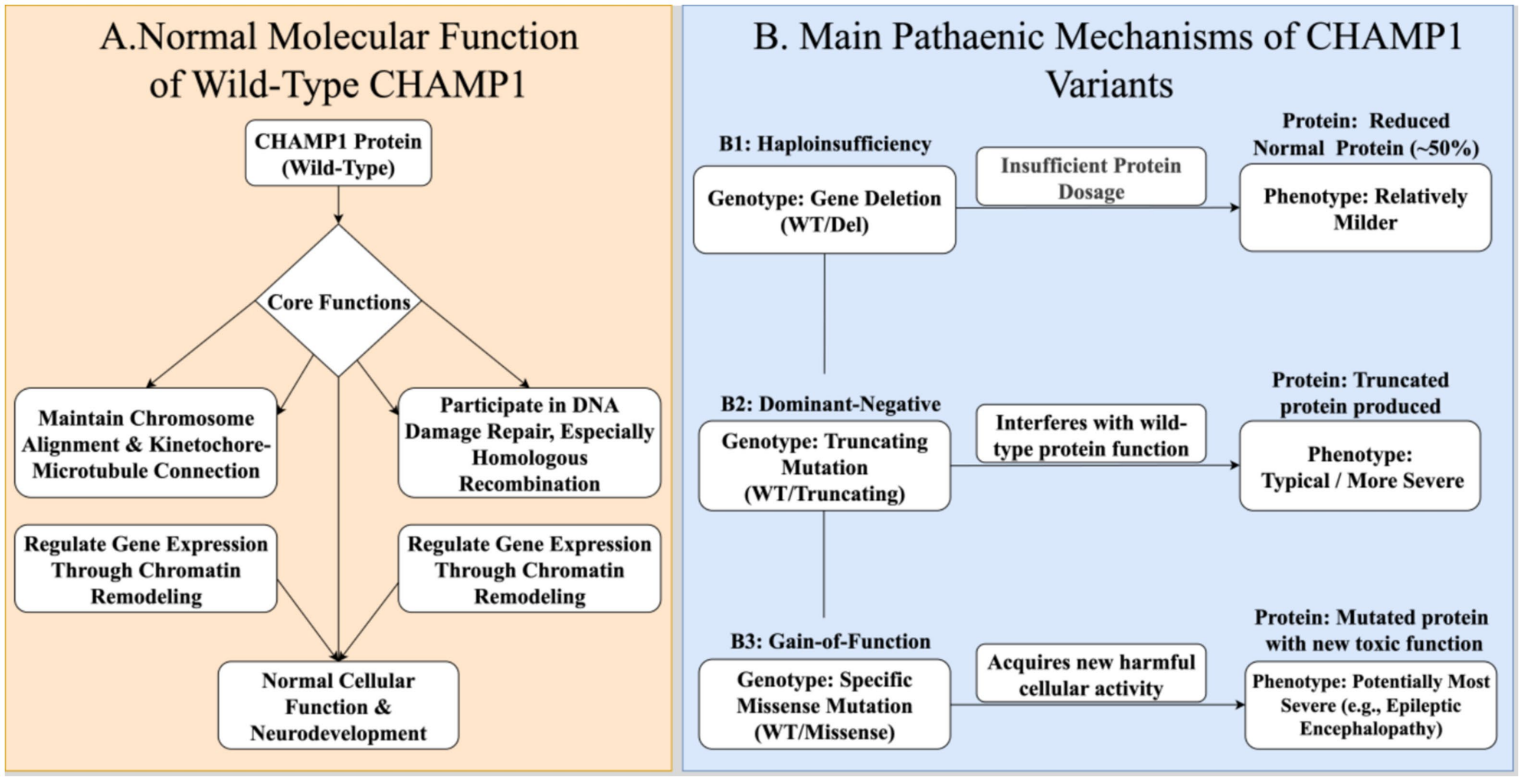
Schematic diagram of the molecular functions of *CHAMP1* protein and pathogenic mechanisms of different variant types. This diagram systematically illustrates the intracellular physiological functions of the *CHAMP1* protein and the pathological mechanisms induced by different gene variants in CHAND/MRD40. **(A)** Normal Molecular Functions of Wild-Type *CHAMP1*. Under normal physiological conditions, the wild-type (WT) *CHAMP1* protein acts as a key regulatory factor in the cell nucleus and exerts three core functions: (1) During mitosis, it precisely regulates the attachment between kinetochores and microtubules to ensure the correct segregation of chromosomes; (2) It participates in the homologous recombination repair pathway of DNA double-strand breaks to maintain genomic integrity; (3) As a component of protein complexes (e.g., the CHAMP complex), it is involved in chromatin remodeling and the regulation of key gene expression. These basic functions collectively ensure the normal physiological activities of cells and are particularly crucial for the complex developmental process of the nervous system. **(B)** Main Pathogenic Mechanisms of *CHAMP1* Variants Heterozygous variants of the *CHAMP1* gene can cause disease through at least three distinct molecular mechanisms, and different mechanisms may correspond to clinical phenotypes of varying severity: (B1) Haploinsufficiency. This mechanism is typically associated with large-fragment deletions involving the *CHAMP1* gene. In this case, the deletion of one allele reduces the total amount of fully functional *CHAMP1* protein to approximately 50% of the normal level. Although this reduction in protein dosage is sufficient to cause disease, the resulting clinical phenotype is usually relatively mild. (B2) Dominant-Negative Effect. This mechanism is mainly associated with truncating mutations (e.g., nonsense mutations or frameshift mutations) that lead to premature protein termination. These mutations not only prevent one allele from producing full-length functional proteins but also result in truncated proteins that may retain partial structural domains. Consequently, these truncated proteins can abnormally bind to or sequester normal proteins or other interacting molecules, thereby interfering with and inhibiting the function of normal *CHAMP1* proteins derived from the wild-type allele. This “disruptive” effect is considered the main cause of more typical and severe clinical phenotypes. (B3) Gain-of-Function. In rare cases, specific missense mutations may endow the *CHAMP1* protein with a novel, cell-toxic function. This abnormally acquired function actively disrupts the normal physiological processes of cells and may lead to the most severe clinical phenotypes, such as early-onset epileptic encephalopathy mentioned in some reports.

For 13q34 microdeletion syndromes involving *CHAMP1*, phenotypes may arise from haploinsufficiency of *CHAMP1* and neighboring genes (e.g., *CDC16*, *UPF3*) (6). Thus, phenotypes of large deletions are more complex, with *CHAMP1* haploinsufficiency contributing to neurodevelopmental aspects but being less severe than intragenic truncating mutations (Figure 1).

## 3 Clinical phenotypes of CHAND

CHANDs (MRD40/CHAND) exhibit a broad and heterogeneous clinical phenotype spectrum, with core features primarily in the nervous system.

### 3.1 Core neurological phenotypes

Global developmental delay and intellectual disability are universal core features of CHAND/MRD40. Almost all reported patients show varying degrees of global developmental delay, with significant delays in motor, cognitive, social, and language domains. Intellectual disability is typically moderate to severe, though some patients (especially those with whole-gene deletions) may exhibit mild intellectual disability or borderline intelligence (10, 11). Language impairment is particularly prominent, with most patients experiencing severe expressive language disorders; some cannot develop meaningful verbal communication or can only utter a few words, while comprehension abilities, though better than expression, remain significantly below normal (7). In motor development, early hypotonia delays gross motor milestones, with some developing later hypertonia or spasticity; fine motor skills are universally impaired, and gait abnormalities are common (7, 10). Many CHAND/MRD40 patients have characteristic facial features (e.g., long face, prominent forehead, thick/arched eyebrows, wide palpebral fissures, broad flat/prominent nasal bridge, long smooth philtrum, thin upper lip), which aid clinical diagnosis (9–11). Behavioral and psychiatric issues, including ASD traits, ADHD, anxiety, aggression, emotional lability, and sleep disorders, are common and impose heavy family burdens (10). Epilepsy is a major neurological complication (incidence: 30–50% or higher), with diverse seizure types; some patients (especially those with specific missense mutations) may develop refractory epilepsy or early-onset epileptic encephalopathy (6, 10, 11). Cranial MRI may show nonspecific structural abnormalities (e.g., ventricular enlargement, corpus callosum dysplasia, white matter changes), though imaging can be normal in some patients (7, 10). Additionally, some CHAND patients develop microcephaly associated with brain hypoplasia (10).

### 3.2 Involvement of other systems

Beyond core neurological manifestations, *CHAMP1* variants may affect multiple organ systems in CHAND/MRD40, with variable incidence and severity. For example, some patients experience intrauterine growth restriction or postnatal growth retardation. Skeletal abnormalities may include finger/toe anomalies (e.g., polydactyly, syndactyly, brachydactyly), scoliosis, and joint laxity/contractures. Ophthalmological issues such as strabismus, refractive errors, nystagmus, and optic nerve hypoplasia have been reported. A minority of patients have congenital heart diseases (e.g., atrial/ventricular septal defects). Genitourinary abnormalities (e.g., cryptorchidism, hydronephrosis, hypospadias) are occasionally observed. Literature also suggests potential immune dysfunction due to repeated infections, though systematic research is lacking. Gastrointestinal issues, a key focus of this review, are discussed in detail below; existing reports mention feeding difficulties, gastroesophageal reflux, and constipation in some patients, though these are not highlighted as core features (10, 11). Notably, some case reports describe recurrent episodes of severe vomiting in CHAND/MRD40 children, particularly after infection or improper diet, which are similar to the presentation of Cyclic Vomitting Syndrome (CVS).

### 3.3 Clinical heterogeneity and genotype–phenotype correlations

CHAND exhibit significant clinical heterogeneity, varying in the severity of core phenotypes (e.g., intellectual disability, developmental delay) and the diversity of accompanying symptoms (e.g., abnormal muscle tone, epilepsy, ophthalmological issues, neurobehavioral problems) (9, 10). Exploring genotype–phenotype correlations is crucial for understanding disease mechanisms and guiding clinical care.

Studies suggest associations between variant types and phenotype severity/characteristics. A key observation is that truncating variants (e.g., nonsense/frameshift mutations, traditionally considered loss-of-function) are linked to more severe phenotypes than *CHAMP1* heterozygous deletions (haploinsufficiency). For example, Levy et al. found that mutation carriers exhibited significantly worse adaptive function (communication, daily living skills, social/motor skills) than deletion carriers, with differences in developmental milestones and comorbidities (11). Amenta et al. proposed that loss-of-function variants (primarily truncating mutations) lead to severe intellectual disability, ASD, and specific facial features, while haploinsufficiency (deletions) causes milder phenotypes (e.g., borderline intelligence) with less impact on quality of life (6, 12).

This genotype–phenotype difference may arise because truncating variants act not via haploinsufficiency alone but through dominant-negative effects or gain-of-function, more severely disrupting cellular physiology in CHAND/MRD40 (6, 11, 13). In contrast, haploinsufficiency from gene deletions—reducing protein levels by half—has milder cellular impacts.

Missense variants represent another genotype–phenotype correlation category. Amenta et al. reported that missense variants may induce more severe phenotypes (e.g., early-onset refractory epilepsy and neuroregression) via gain-of-function mechanisms. However, limited reports exist on their pathogenicity and phenotype spectra, requiring further clarification.

In summary, clinical heterogeneity in *CHAMP1*-related disorders stems partly from genetic heterogeneity. Different variant types (truncating mutations, deletions, missense mutations) and corresponding mechanisms (dominant-negative effects, haploinsufficiency, gain-of-function) shape diverse phenotype spectra. While current genotype–phenotype insights are evolving, existing findings highlight the importance of variant-type differentiation in clinical practice. Larger patient cohorts, systematic phenotyping, and molecular function studies will refine these correlations for precise genetic counseling, prognosis, and personalized interventions (11) (Table 1).

**Table 1:** Summary table of associations between different *CHAMP1* gene variant types and clinical phenotypes.

Variant type	Typical variant examples	Hypothesized molecular mechanism	Severity and characteristics of core neurophenotypes	Incidence and characteristics of systemic phenotypes (Including gastrointestinal symptoms)	Literature source
Truncating Mutation	Nonsense mutations (e.g., p. Y636*), frameshift mutations	Mainly dominant negative (DN) effect, partially accompanied by haploinsufficiency	1. Global Developmental Delay (GDD): Present in 100% of patients, with significant delays in motor, cognitive, and language domains; 2. Intellectual Disability (ID): Almost all moderate to severe, greatly affecting quality of life; 3. Language Disorder: Severe expressive disorder, most patients cannot develop meaningful speech; 4. Neurobehavioral Abnormalities: High incidence of autism spectrum disorder (ASD) traits and attention - deficit/hyperactivity disorder (ADHD); 5. Epilepsy: Incidence is 30–50% or higher, with diverse seizure types and no tendency to be refractory; 6. Brain Structural Abnormalities: Relatively high incidence of nonspecific abnormalities such as enlarged ventricles and corpus callosum dysgenesis	1. Growth Abnormalities: Some patients have postnatal growth retardation; 2. Skeletal/Ophthalmic Abnormalities: More sporadic cases of digital deformities, strabismus, etc.; 3. Gastrointestinal Symptoms: High incidence of feeding difficulties (70% in reference cohort data), frequent constipation, no widespread reports of recurrent vomiting (only 1 case with p. Y636* variant was observed to have vomiting similar to cyclic vomiting syndrome (CVS))	(6, 9–11, 14)
Gene Deletion	13q34 microdeletion (including *CHAMP1* and adjacent genes such as *CDC16*, *UPF3*)	Haploinsufficiency	1. Global Developmental Delay (GDD): Present in 100% of patients, but less severe; 2. Intellectual Disability (ID): Mostly mild or borderline intellectual disability, only a few affect daily functions; 3. Language Disorder: Mild to moderate expressive delay, comprehension ability close to normal; 4. Neurobehavioral Abnormalities: Incidence of ASD/ADHD traits is lower than that in patients with truncating mutations; 5. Epilepsy: Incidence is lower than that in patients with truncating mutations, mostly occasional and easily controllable; 6. Brain Structural Abnormalities: High proportion of normal imaging results, and abnormalities are mostly mild ventricular enlargement	1. Growth Abnormalities: Low incidence of intrauterine growth restriction or postnatal growth retardation; 2. Skeletal/Ophthalmic/Cardiac Abnormalities: Rare, only very individual cases reported; 3. Gastrointestinal Symptoms: Low incidence of feeding difficulties (significantly lower than 70%), occasional constipation, no severe gastrointestinal complications requiring medical intervention (such as gastrostomy tube)	(6, 11, 12)
Missense Mutation	Point mutations targeting zinc - finger domains, phosphorylation sites	Mainly gain-of-function (GOF)	1. Global Developmental Delay (GDD): Present in 100% of patients, extremely severe, with severe lag in neurodevelopmental milestones; 2. Intellectual Disability (ID): All severe to profound, no independent living ability; 3. Epilepsy: Characterized by early - onset refractory epileptic encephalopathy, frequent seizures with poor response to drugs, accompanied by neurodegeneration; 4. Brain Structural Abnormalities: High incidence of brain dysplasia and white matter changes, and the degree is severe	1. Growth Abnormalities: High incidence of intrauterine growth restriction, mostly accompanied by severe postnatal growth retardation; 2. Multisystem Abnormalities: Incidence of rare complications such as congenital heart disease (such as ventricular septal defect) and genitourinary abnormalities (such as cryptorchidism) is higher than that of other variant types; 3. Gastrointestinal Symptoms: No clear cohort data, only sporadic cases mention mild feeding difficulties, no reports of constipation or vomiting	(6)
Rare Variants with Unclear Classification (such as small-fragment insertions/deletions, splice site mutations)	Splice site mutations	Mostly haploinsufficiency or mild dominant negative effect	Phenotypes are between “truncating mutations” and “gene deletions”: 1. Global Developmental Delay (GDD): Moderate delay; 2. Intellectual Disability (ID): Moderate, partial daily assistance required; 3. Epilepsy: Incidence is about 20–30%, easily controllable	Low incidence of systemic phenotypes, only a few cases report mild skeletal abnormalities (such as joint laxity), and gastrointestinal symptoms are mainly mild feeding difficulties, with no severe complications	(10, 11)

## 4 Association between *CHAMP1* gene variants and gastrointestinal symptoms

### 4.1 Existing reports on gastrointestinal symptoms in *CHAMP1*-related disorders

Gastrointestinal (GI) symptoms are emerging as common comorbidities in *CHAMP1*-related disorders. Garrity et al. (14) systematically analyzed 43 individuals with *CHAMP1* variants (13 new cases + 30 literature cases of CHAND/MRD40) and found GI symptoms in a high proportion: 70% had feeding difficulties, with severe cases requiring long-term gastrostomy tube feeding for growth maintenance. Constipation was also a frequent symptom (14). Phenotypic analysis of White-Sutton syndrome (caused by *POGZ* variants, a *CHAMP1*-interacting protein) showed high rates of GI abnormalities (functional/structural), indirectly suggesting that *CHAMP1-POGZ* complex integrity is critical for GI function in CHAND/MRD40 (15). Despite growing attention, systematic large-cohort studies on the incidence and clinical characteristics of specific GI symptoms (e.g., vomiting, gastroesophageal reflux) and their association with *CHAMP1* variants are lacking (Table 2).

**Table 2:** Summary table of gastrointestinal symptoms reported in literature on CHAND/MRD40.

Sample size (Cases)	Type of gastrointestinal symptom	Specific manifestations	Incidence/Reporting status	Key notes	Literature source
43 cases (include 13 new cases + 30 literature cases)	Feeding difficulties, constipation	70% of patients had feeding difficulties; some required long-term gastrostomy tube feeding to maintain growth; constipation was a common symptom (specific proportion not specified).	Feeding difficulties: 70%; Constipation: Specific value not specified, but clearly stated as a “frequently occurring” symptom	Based on large-sample data from systematic analysis, it is one of the most definitive literatures on the incidence of gastrointestinal symptoms in CHAND/MRD40; the mentioned feeding difficulties were mostly severe and required medical intervention.	(14)
Not specified (focused on variant-phenotype association, no separate statistics on gastrointestinal symptoms)	Not specified	Only mentioned that “multisystem involvement may include gastrointestinal problems” without elaborating on specific symptoms.	Incidence not specified	Focused on molecular mechanisms and genotype–phenotype associations; gastrointestinal symptoms were only mentioned secondarily with no detailed data.	(6)
Not specified (cohort for neurobehavioral phenotype study)	Not specified	Mentioned that “multisystem complications may include gastrointestinal dysfunction” without listing specific symptoms.	Incidence not specified	Research focused on neurobehavioral symptoms such as autism spectrum disorder (ASD) and attention deficit hyperactivity disorder (ADHD); gastrointestinal symptoms were only a general description of multisystem involvement.	(10)
Not specified (cohort for genotype–phenotype comparison)	Not specified	When comparing phenotypes between patients with truncating mutations and gene deletions, mentioned that “multisystem symptoms may differ” but did not specifically mention gastrointestinal symptoms.	Incidence not specified	Core focus was on demonstrating phenotypic severity differences between truncating mutations and gene deletions; no details on gastrointestinal symptoms were involved.	(11)
1 cases (first Chinese case with *CHAMP1* frameshift mutation)	No definite gastrointestinal symptoms reported	Only described neurodevelopmental symptoms (e.g., severe intellectual disability, language developmental delay); no gastrointestinal-related manifestations were mentioned.	-	Single-case report; no signs of gastrointestinal involvement were found, which may be attributed to individual case differences.	(12)
1 cases (*CHAMP1* p. Y636 variant)	No definite gastrointestinal symptoms reported	Focused on gene variant verification and neurodevelopmental phenotypes (e.g., global developmental delay, distinctive facial features); no gastrointestinal symptoms were mentioned.	-	Single-case report; no gastrointestinal symptoms were observed, which may be related to variant type or individual differences.	(9)
Not specified (cohort for POGZ-related White-Sutton syndrome)	Gastrointestinal abnormalities (indirect association)	Reported a “high proportion of gastrointestinal abnormalities (functional/structural)” in patients with POGZ gene variants; since POGZ is an interacting protein of *CHAMP1*, it indirectly suggests that *CHAMP1*-POGZ complex abnormalities may affect gastrointestinal function.	“High proportion” of gastrointestinal abnormalities in POGZ variant patients; specific value not specified	Not a direct study on *CHAMP1* cases, but provides mechanistic reference for gastrointestinal symptoms in CHAND/MRD40 based on protein–protein interaction.	(7, 15)

### 4.2 Dual mechanism hypothesis for gastrointestinal symptoms caused by *CHAMP1* functional defects in CHAND/MRD40

The pathophysiological mechanisms underlying gastrointestinal (GI) dysfunction caused by *CHAMP1* gene variants may not stem from a single pathway, but rather a complex process involving the interweaving of local and central, structural and functional factors. We propose a dual mechanism hypothesis, suggesting that intrinsic developmental defects of the Enteric Nervous System (ENS) serve as the structural basis for GI symptoms, while dysregulation of the Brain-Gut Axis acts as a symptom amplifier and trigger on this basis.

#### 4.2.1 Intrinsic ENS defects: the structural and functional basis of GI dysfunction in CHAND/MRD40

Known as the “brain” of the gastrointestinal tract, the ENS relies on the integrity of its network and the maturity of its function to maintain normal GI motility. The formation of the ENS involves large-scale proliferation, directed migration, and precise differentiation of neural crest stem cells—processes that are highly dependent on the precise regulation of the cell cycle and genomic stability, which are core functional domains of the *CHAMP1* protein (1). Therefore, functional defects of *CHAMP1* are likely to impair the normal development of the ENS at the source, leading to: (1) insufficient numbers of intestinal neurons or glial cells; (2) sparse or abnormal connections in the neuronal network; (3) impaired synthesis and release of neurotransmitters. These intrinsic ENS defects can directly cause disordered contraction rhythms and weakened propulsion of gastrointestinal smooth muscles, manifesting as symptoms such as chronic constipation, delayed gastric emptying, and feeding difficulties.

#### 4.2.2 Dysregulation of the brain-gut axis: amplification and triggering of GI symptoms in CHAND/MRD40

The brain-gut axis is a bidirectional information pathway connecting the central nervous system (CNS) and the gastrointestinal tract. *CHAMP1* variants first induce extensive developmental abnormalities of the CNS, which inevitably disrupt the normal function of the brain-gut axis. On one hand, central descending regulation of the gastrointestinal tract becomes disordered. For example, dysfunction of the autonomic nervous system may lead to abnormal vagal tone or excessive sympathetic activation, thereby altering GI motility patterns and sensitivity. On the other hand, *CHAMP1* patients often exhibit comorbidities such as anxiety and abnormal sensory processing (10), which lower their tolerance threshold to stressors (e.g., infections, emotional fluctuations). These stress signals are amplified through the dysregulated brain-gut axis, easily triggering or exacerbating GI symptoms—consistent with our clinical observation that recurrent vomiting in some children is closely associated with factors like infections, providing a potential explanation for the emergence of cyclic vomiting syndrome (CVS)-like phenotypes.

In summary, we hypothesize that the occurrence of *CHAMP1*-related GI symptoms results from the combined effects of ENS hypoplasia and central regulatory failure. Defects in the ENS establish the “hardware” basis of gastrointestinal functional fragility, while brain-gut axis dysregulation provides the “software” triggers of functional impairment; together, they determine the occurrence and severity of GI symptoms in patients. This dual mechanism hypothesis provides a theoretical framework for understanding the complex phenotypes of the disease and suggests that future treatments may need to simultaneously focus on improving local intestinal function and regulating central nervous system status.

### 4.3 *CHAMP1* and recurrent vomiting: clinical observations and future research directions

Some clinical cases of CHAND/MRD40 children with *CHAMP1* gene variants (including truncated mutations such as p. Y636*) are characterized by recurrent episodes of severe vomiting associated with specific triggers (such as infection, improper diet), and this pattern of vomiting suggests the possibility of clinical manifestations similar to cyclic vomiting syndrome (CVS). This vomiting pattern suggests a clinical phenotype resembling cyclic vomiting syndrome (CVS). CVS is a functional gastrointestinal disorder characterized by stereotypical (similar features in each episode), intense nausea and vomiting, with complete symptom resolution between episodes. Its etiology and pathogenesis remain incompletely understood. While no direct literature links *CHAMP1* variants to typical CVS, whole-exome/genome sequencing studies by Bar et al. identified multiple candidate genes related to ion transport or energy metabolism in CVS patients, implicating intracellular cation homeostasis imbalance and mitochondrial dysfunction in its pathogenesis (16). This provides a theoretical basis for exploring potential mechanisms underlying *CHAMP1*-associated vomiting.

Given the implication of mitochondrial dysfunction in CVS pathogenesis (16), and *CHAMP1*’s role in regulating nuclear gene expression, it is plausible that *CHAMP1* deficiency could indirectly impact mitochondrial bioenergetics or ion channel expression, and the inherent autonomic nervous system instability and stress sensitivity in neurodevelopmental disorders, *CHAMP1* variants may increase susceptibility to CVS-like vomiting by perturbing pathways implicated in CVS. For example, whether *CHAMP1* dysfunction indirectly impairs mitochondrial bioenergetics or disrupts key ion channel expression/function (though direct evidence is lacking), thereby lowering the threshold for vomiting reflexes or dysregulating autonomic control, warrants further investigation. Additionally, comorbid anxiety and sensory processing abnormalities common in *CHAMP1*-related disorders (10) may act as triggers or exacerbating factors for CVS-like episodes.

Future research on CHAND/MRD40 should focus on: (1) *Large-scale cohort studies*: Systematically document gastrointestinal symptoms, particularly vomiting characteristics (frequency, pattern, triggers, duration, severity, associated symptoms, treatment response) in *CHAMP1* variant patients. Compare these phenotypes rigorously against CVS diagnostic criteria (e.g., Rome IV) to determine overlap and specificity. (2) *Basic functional investigations*: Utilize patient-derived induced pluripotent stem cell (induced Pluripotent Stem Cells, iPSCs)-differentiated neurons or intestinal organoids, alongside *CHAMP1* gene-edited animal models, to elucidate how *CHAMP1* variants affect enteric nervous system development, autonomic function, mitochondrial metabolism, and ion channel/signaling pathways relevant to CVS. (3) *Biomarker discovery*: Explore potential biomarkers, such as neuroimaging changes, autonomic function metrics, or serum/cerebrospinal fluid metabolites/inflammatory factors, to identify *CHAMP1* subpopulations with CVS-like phenotypes and guide personalized therapy. (4) *Targeted therapeutic strategies*: Based on mechanistic insights, cautiously evaluate and develop specific interventions for *CHAMP1*-related vomiting, including lifestyle modifications, pharmacologic interventions (e.g., antimigraine medications, antiemetics, or mitochondrial function modifiers used in CVS under careful risk–benefit assessment), and rehabilitative approaches like behavioral and sensory integration training to improve patient quality of life.

### 4.4 Genotype–phenotype associations in gastrointestinal symptoms

Current evidence limitations and future directions while preliminary observations in neurological phenotypes suggest that truncation mutations and large deletions of the *CHAMP1* gene may lead to varying clinical outcomes in CHAND/MRD40, indicating potential genotype–phenotype associations (6), whether this correlation extends to the gastrointestinal (GI) system remains an unresolved question. A systematic review of existing literature reveals that although GI symptoms such as feeding difficulties and constipation are common in *CHAMP1*-related disorders (14), no studies have definitively established a direct link between specific variant types (e.g., truncation or misreadings) or their genomic locations and the risk or severity of particular GI symptoms (e.g., vomiting or constipation). The primary reasons for this status quo lie in the predominance of small-scale analyses or case reports, coupled with qualitative rather than quantitative descriptions of GI symptoms and the lack of standardized evaluation systems. These factors make cross-study data integration and comparative analysis extremely challenging, hindering the identification of reliable association trends. Similarly, critical clinical evidence supporting the “brain-gut axis” dysfunction hypothesis—such as whether the severity of neurological impairments parallels the severity of GI symptoms—remains unverifiable due to similar data limitations. Therefore, it is crucial to clarify the current evidence gaps in GI symptom genotypic-phenotypic associations. Future research should prioritize establishing an international multicenter collaborative patient registry system with standardized evaluation criteria, enabling prospective and systematic data collection across multiple organ systems including neurological and digestive systems. By integrating large-scale, high-quality data, we aim to reveal precise correlations between *CHAMP1* gene variants and gastrointestinal dysfunction. This will provide critical evidence for elucidating pathophysiological mechanisms of CHAND/MRD40, guiding clinical prognosis assessment, and exploring potential personalized intervention strategies.

## 5 Summary and outlook

Heterozygous variants of the *CHAMP1* gene, particularly *de novo* truncating mutations, are the confirmed molecular etiology of MRD40/CHAND characterized by intellectual disability, global developmental delay, language impairment, and specific facial features. As a key nuclear protein maintaining chromosome alignment and genomic stability during cell mitosis, the functional diversity of *CHAMP1* determines the broad spectrum of clinical phenotypes caused by its mutations. In addition to core manifestations in the central nervous system, such as cognitive and motor dysfunction, epilepsy, and behavioral abnormalities, involvement of other systems has gradually been recognized—particularly gastrointestinal symptoms highlighted in this review, including feeding difficulties, constipation, and cyclic vomiting syndrome (CVS)-like manifestations in some patients, all of which significantly impact quality of life. Current understanding of the pathogenic mechanisms of *CHAMP1* variants remains incomplete, with coexisting hypotheses of haploinsufficiency, dominant-negative effects, or gain-of-function, indicating that different types of gene variants may lead to diverse clinical consequences through distinct molecular pathways.

Despite significant progress in understanding *CHAMP1*-related diseases in recent years, numerous challenges remain, while also indicating directions for future research. Moving forward, in-depth exploration should focus on the following aspects:

### 5.1 Deepening exploration and elucidation of molecular mechanisms

Current knowledge regarding how *CHAMP1* gene variants precisely affect cellular functions and physiological processes in different tissues and organs—especially their specific mechanisms of action outside the nervous system (e.g., the gastrointestinal tract)—remains limited. Future research should strive to:

(a) Clarify the specific molecular pathological mechanisms corresponding to different variant types (truncating, missense, deletions), distinguishing whether they result from protein function loss, dosage insufficiency, or the production of new proteins with dominant-negative effects or gain-of-function (potentially with toxic effects).
(b) Investigate in detail how protein complexes formed by *CHAMP1* and its interacting proteins (such as *POGZ*, *SETDB1*, etc.) synergize in key cellular processes such as chromatin remodeling, gene expression regulation, and DNA damage repair, and how disruptions in these processes specifically affect the development, differentiation, and function of different cell types, including neurons and intestinal cells.
(c) Utilize multi-omics technologies (such as single-cell transcriptomics, proteomics, and metabolomics) to integratively analyze patient-derived samples and disease models, systematically mapping the molecular network dysregulation caused by *CHAMP1* functional deficiency, with the aim of discovering new biological markers and potential therapeutic targets.

### 5.2 Systematic clinical research and precise depiction of phenotype spectra

The clinical heterogeneity of *CHAMP1-*related diseases poses challenges for diagnosis and management. Urgent needs include:

(a) Establishing internationally collaborative, standardized clinical cohorts and biobanks for *CHAMP1* patients, with detailed longitudinal data recording multi-system manifestations including neurodevelopment, behavior, psychology, motor function, epilepsy, and gastrointestinal, immune, and endocrine systems, to enable more refined genotype–phenotype correlation analyses and identify potential phenotypic subgroups and prognostic factors.
(b) Developing and validating specific clinical assessment tools and outcome indicators for *CHAMP1*-related disorders to more accurately quantify disease burden and evaluate intervention effects.
(c) Strengthening research on non-classical phenotypes and disease natural history, particularly the incidence, severity, pathophysiological mechanisms of gastrointestinal symptoms (such as feeding difficulties, constipation, and repeated vomiting), and their comprehensive impact on children’s growth and quality of life, to clarify whether they constitute core or common accompanying features of this syndrome.

### 5.3 Research, development, and translation of innovative treatment strategies

Current treatments for *CHAMP1*-related diseases primarily rely on symptomatic support and rehabilitation training, with a lack of specific therapies targeting the etiology.

(a) Based on an in-depth understanding of molecular mechanisms, explore potential targeted treatment strategies—for example, developing small-molecule drugs or nucleic acid drugs (such as Antisense Oligonucleotide, ASO) to inhibit the expression or function of mutant proteins for dominant-negative effect mutations; exploring corresponding modulators for disorders in specific signaling pathways. Drug repurposing is also worth attempting.
(b) In the long term, gene therapy (such as gene replacement or gene editing) represents a potential cure for monogenic diseases. Despite significant technical challenges, continued attention to and investment in basic research are necessary.
(c) Optimize existing multidisciplinary comprehensive management programs to provide more personalized and precise interventions for patients’ cognitive, language, motor, behavioral, epileptic, and gastrointestinal issues. For example, for children with CVS-like vomiting, draw on CVS diagnostic and treatment experience to explore comprehensive programs including dietary management, drug prevention, and acute-phase treatment.

### 5.4 Building a patient-centered omnidirectional support system

As a rare and complex chronic disease, *CHAMP1*-related disorders impose heavy physical, psychological, and economic burdens on children and their families.

(a) Enhance clinicians’ awareness of the disease to facilitate early diagnosis and provide timely and accurate genetic counseling and reproductive guidance for diagnosed families.
(b) Establish and improve a collaborative diagnosis and treatment network composed of multidisciplinary experts, including neurologists, gastroenterologists, developmental behavioral pediatricians, rehabilitation specialists, nutritionists, and genetic counselors, to provide standardized management and care throughout the patient’s lifecycle.
(c) Strongly support the establishment and development of patient organizations to promote experience sharing, emotional support, and resource sharing among patient families, jointly enhancing care capabilities and improving the overall quality of life for patients and their families.

In summary, through close collaboration across multiple disciplines and integration of breakthroughs in basic research with innovations in clinical practice, we aspire to comprehensively and deeply understand the pathogenic mechanisms and clinical spectra of *CHAMP1* gene variants in the future, continuously optimize diagnostic and management strategies, and ultimately bring new hope for improving the prognosis and quality of life for children with CHANDs and their families.
